# In the national epidemiological bulletins – a selection from recent issues

**DOI:** 10.2807/1560-7917.ES.2019.24.50.1912121

**Published:** 2019-12-12

**Authors:** Eurosurveillance editorial team

**Affiliations:** 1 European Centre for Disease Prevention and Control (ECDC), Stockholm, Sweden

**Keywords:** infectious diseases, public health, Europe

**Healthcare-associated infections and antibiotic resistance**

 

Prolonged non-specific disease in cardiac valve-operated people: *Mycobacterium chimaera* infection associated with the use of heater-cooler equipment is a novel and rare, but important differential diagnosis

EPI-NEWS 40, 2019

25 October 2019, Denmark

https://en.ssi.dk/news/epi-news/2019/no-40---2019

 

Frequency, characteristics and distribution of MRSA in Germany: situation for 2017–2018

Epidemiologisches Bulletin 42/2019

17 October 2019, Germany

https://www.rki.de/DE/Content/Infekt/EpidBull/Archiv/2019/Ausgaben/42_19.pdf?__blob=publicationFile

 

Suspected and laboratory confirmed reported norovirus outbreaks in hospitals (England) and norovirus laboratory reports (England and Wales): weeks 36 to 39, 2019

Health Protection Report; 13(36)

11 October 2019, United Kingdom

https://assets.publishing.service.gov.uk/government/uploads/system/uploads/attachment_data/file/838807/hpr3619_noro.pdf

 

Information about the occurrence of *Klebsiella pneumoniae* with OXA-48 and NDM-1 carbapenemases and colistin resistance in Mecklenburg-Vorpommern

Epidemiologisches Bulletin 40/2019

2 October 2019, Germany

https://www.rki.de/DE/Content/Infekt/EpidBull/Archiv/2019/Ausgaben/40_19.pdf?__blob=publicationFile

 

**Food- and waterborne diseases**

 

Prolonged local *Listeria monocytogenes* outbreak resolved

EPI-NEWS 45, 2019

20 November 2019, Denmark

https://en.ssi.dk/news/epi-news/2019/no-45---2019

 

Routine reports of gastrointestinal infections in humans, England and Wales: September and October 2019

Health Protection Report; 13(40)

8 November 2019, United Kingdom

https://assets.publishing.service.gov.uk/government/uploads/system/uploads/attachment_data/file/845643/hpr4019_GIs.pdf

 

Listeriosis outbreak with *Listeria monocytogenes* sequence cluster type 2521 (Sigma1) in Germany

Epidemiologisches Bulletin 41/2019

10 October 2019, Germany

https://www.rki.de/DE/Content/Infekt/EpidBull/Archiv/2019/Ausgaben/41_19.pdf?__blob=publicationFile

 

Increase in typhoid notifications in travellers returning from Pakistan

Epi-Insight 2019;20(10)

October 2019, Ireland

http://ndsc.newsweaver.ie/epiinsight/1npbbpsg5wm?a=1&p=55721182&t=17517774

 

**Hepatitides**

 

Acute hepatitis B: national enhanced surveillance report January to June 2019

Health Protection Report; 13(38)

25 October 2019, United Kingdom

https://assets.publishing.service.gov.uk/government/uploads/system/uploads/attachment_data/file/841889/hpr3819_ct-hepB.pdf

 

Special edition: National day against viral hepatitis, 2019

Bulletin épidémiologique hebdomadaire, 24-25/2019

24 September2019, France

http://beh.santepubliquefrance.fr/beh/2019/24-25/index.html

 

**Vaccine-preventable diseases**

 

Pertussis vaccination programme for pregnant women update: vaccine coverage in England, April to September 2019

Health Protection Report; 13(41)

2 December 2019, United Kingdom

https://assets.publishing.service.gov.uk/government/uploads/system/uploads/attachment_data/file/849023/hpr4119_AA_prntl-prtsss-vc.pdf

 

Invasive meningococcal disease in England: annual laboratory confirmed reports for epidemiological year 2018 to 2019

Health Protection Report; 13(38)

29 October 2019, United Kingdom

https://assets.publishing.service.gov.uk/government/uploads/system/uploads/attachment_data/file/842368/hpr3819_IMD-ann.pdf

 

Meningococcal disease 2018

EPI-NEWS 40, 2019

25 October 2019, Denmark

https://en.ssi.dk/news/epi-news/2019/no-40---2019

 

Nationwide whooping cough epidemic

EPI-NEWS 38, 2019

8 October 2019, Denmark

https://en.ssi.dk/news/epi-news/2019/no-38---2019

 

Laboratory confirmed cases of pertussis (England): April to June 2019

Health Protection Report; 13(34)

30 September 2019, United Kingdom

https://assets.publishing.service.gov.uk/government/uploads/system/uploads/attachment_data/file/835104/hpr3419_prtsss.pdf

 

Investigation of the Bavarian State Office for Health and Food Safety on tuberculosis among asylum seekers in Bavaria

Epidemiologisches Bulletin 39/2019

26 September 2019, Germany

https://www.rki.de/DE/Content/Infekt/EpidBull/Archiv/2019/Ausgaben/39_19.pdf?__blob=publicationFile

 

**Respiratory diseases**

 

Legionellosis in the spotlight

Epi-Insight 2019;20(11)

November 2019, Ireland

http://ndsc.newsweaver.ie/epiinsight/16vaazyleppimcmkeer4wk?a=1&p=55896984&t=17517774

 

Focused issue: Influenza

Bulletin épidémiologique hebdomadaire, 28/2019

21 October 2019, France

http://beh.santepubliquefrance.fr/beh/2019/28/index.html

 

**Sexually transmitted diseases**

 

HIV studies and projects at the Robert Koch Institute

Epidemiologisches Bulletin 49/2019

5 December 2019, Germany

https://www.rki.de/DE/Content/Infekt/EpidBull/Archiv/2019/Ausgaben/49_19.pdf?__blob=publicationFile

 

Special edition: Epidemiological situation and screening for HIV and other STIs

Bulletin épidémiologique hebdomadaire, 31-32/2019

26 November 2019, France

http://beh.santepubliquefrance.fr/beh/2019/31-32/index.html

 

HIV 2018

EPI-NEWS 44, 2019

20 November 2019, Denmark

https://en.ssi.dk/news/epi-news/2019/no-44---2019

 

Estimated number of new HIV infections and the total number people living with HIV in Germany, status at the end of 2018

Epidemiologisches Bulletin 46/2019

14 November 2019, Germany

https://www.rki.de/DE/Content/Infekt/EpidBull/Archiv/2019/Ausgaben/46_19.pdf?__blob=publicationFile

 

Monitoring community HIV testing in Ireland

Epi-Insight 2019;20(11)

November 2019, Ireland

http://ndsc.newsweaver.ie/epiinsight/12tu3c2wui1imcmkeer4wk?a=1&p=55896983&t=17517774

 

Chlamydia 2018

EPI-NEWS 38, 2019

8 October 2019, Denmark

https://en.ssi.dk/news/epi-news/2019/no-38---2019

 

**Zoonoses and vector-borne diseases**

 

New autochthonous case of West Nile virus infection has been confirmed

Epidemiologisches Bulletin 44/2019

31 October 2019, Germany

https://www.rki.de/DE/Content/Infekt/EpidBull/Archiv/2019/Ausgaben/44_19.pdf?__blob=publicationFile

First case of West Nile virus infection transmitted by mosquitoes in Germany

Epidemiologisches Bulletin 40/2019

2 October 2019, Germany

https://www.rki.de/DE/Content/Infekt/EpidBull/Archiv/2019/Ausgaben/40_19.pdf?__blob=publicationFile

 

**Other**

 

Travel-associated diseases 2018

Epidemiologisches Bulletin 48/2019

28 November 2019, Germany

https://www.rki.de/DE/Content/Infekt/EpidBull/Archiv/2019/Ausgaben/48_19.pdf?__blob=publicationFile

 

Laboratory surveillance of pyogenic and non-pyogenic streptococcal bacteraemia in England, Wales and Northern Ireland: 2018

Health Protection Report; 13(41)

20 November 2019, United Kingdom

https://assets.publishing.service.gov.uk/government/uploads/system/uploads/attachment_data/file/847147/hpr4119_pnp-strptccc.pdf

 

Occurrence of enterovirus D68

EPI-NEWS 39, 2019

10 October 2019, Denmark

https://en.ssi.dk/news/epi-news/2019/no-39---2019 

